# Prevalence and epidemiological determinants of metabolically obese but normal-weight in Chinese population

**DOI:** 10.1186/s12889-020-08630-8

**Published:** 2020-04-15

**Authors:** Qianqian Zheng, Weihua Lin, Chengguo Liu, Yaohan Zhou, Tianhui Chen, Liqun Zhang, Xuhui Zhang, Senhai Yu, Qiong Wu, Ziqi Jin, Yimin Zhu

**Affiliations:** 1 Department of Epidemiology & Biostatistics, School of Public Health, Zhejiang University, Hangzhou, 310058 Zhejiang China; 2 Hangzhou MetaWell Technology Co., Hangzhou, China; 3 Putuo District People’s Hospital, Zhoushan, 316100 Zhejiang China; 4 Department of Cancer Prevention, Zhejiang Cancer Hospital, Hangzhou, 310022 Zhejiang Province China; 5 Hangzhou Center for Disease Control and Preventio, Hangzhou, 310051 Zhejiang China; 6 Daicun Town Community Health Service Center, Xiaoshan District, Hangzhou, Zhejiang China; 7 Department of Respiratory Diseases, Sir Run Run Shaw Hospital Affiliated to School of Medicine, Zhejiang University, Hangzhou, 310020 Zhejiang China

**Keywords:** Metabolically obesity normal-weight (MONW), Heterogeneity, Body mass index (BMI), Waist circumference (WC)

## Abstract

**Background:**

There is metabolic heterogeneity in normal-weight individuals, however, there has been limited research in the Chinese population. This study aimed to investigate the prevalence, distribution and epidemiological determinants of metabolically obese but normal-weight (MONW) in a Chinese population.

**Methods:**

A total of 17,876 normal-weight individuals were recruited from 37,815 individuals in Zhejiang province in southeastern China. Normal-weight was defined as a body mass index (BMI) of 18.5–23.9 kg/m^2^. Metabolically abnormal traits were assessed by metabolic syndrome criteria from the International Diabetes Federation (IDF) in 2015. MONW was defined as individuals who had at least two metabolically abnormal trait but normal weight. Multiple logistic regression was used to investigate MONW risk factors, adjusting for potential confounders.

**Results:**

The prevalence of metabolic abnormality was 34.1% in normal-weight individuals, and the overall prevalence of MONW was 16.1% in the general population. Different MONW distributions were found between men and women depending on age. Compared with women, men had a significantly higher MONW prevalence among those aged < 45 years old, and there was a lower prevalence for those aged ≥50 years old. Higher BMI or waist circumference (WC), central obesity, menopause, and family histories of hypertension, diabetes, and cardiovascular diseases, increased MONW risk. Higher education levels, regular alcohol drinking, and balanced or vegetarian food preferences reduced MONW risk.

**Conclusions:**

Normal-weight individuals have metabolic heterogeneity in China. The MONW distribution between men and women depends on age. BMI, WC, dietary factors, and family history of chronic diseases, are associated with metabolic status.

## Background

Obesity, characterized by excessive accumulation and storage of body fat, is a cluster of chronic metabolic disorders such as hypertension, hyperglycemia, dyslipidemia, and insulin resistance. It is well-known that obesity increases the risk of type 2 diabetes, coronary heart disease (CHD), stroke, and some cancers [1]. The global obesity prevalence has dramatically increased, almost tripling since 1975 [2]. Consequently, obesity has become a severe public health issue [3]. Theoretically, the prevention and control of obesity is key to controlling the epidemics of common chronic diseases. Previous studies have indicated that obesity is a heterogeneous disease in terms of body shapes (“apple” and “pear” shapes), pathologic types of lipocytes (hyperplasia and hypertrophy) and metabolic status (metabolically normal obesity and metabolically abnormal obesity) [4]. Obese individuals with an “apple shape,” hypertrophy, or metabolic abnormality have higher risk for adverse outcomes [4, 5].

On the other hand, not all individuals with normal-weight are metabolically normal. Some normal-weight individuals also have multiple cardio-metabolic disorders such as insulin resistance, and high levels of inflammation, triglycerides (TG), blood pressure (BP), fasting plasma glucose (FPG), and decreased high-density lipoprotein cholesterol (HDL-C) [4], These individuals are typically referred to as metabolically obese but normal-weight (MONW) [6, 7]. Kramer, et al. [8] reported that MONW was associated with an increased risk of cardiovascular disease and all-cause mortality. From a public health perspective, due to having a normal body mass index (BMI), MONW individuals are easily masked by the need for screening, thus may be paid little attention to prevention, and delay the diagnosis and treatment [6, 9–12]. Therefore, recently, MONW has attracted considerable attention.

Previous studies revealed that the global prevalence of MONW varied from 5 to 45% [4, 13]. This variation may be due to individuals’ age, gender, ethnicity, and geographic location, and variation in MONW definitions (i.e., different criteria on obesity and metabolic abnormality). Previous studies mainly focused on Caucasians and people of African descent; research in this space on Asians is very limited. In 2017, Zhang et al. [14] reported that metabolic syndrome prevalence was 8.14% among normal-weight individuals in Beijing, China. However, individuals in this study were recruited from a health examination center, and this may have led to selection bias due to the low representativeness of these individuals; hence, the results might be difficult to extrapolate. Additionally, the determinants of metabolic heterogeneity in normal-weight individuals remain unknown.

In this study, we aimed to investigate MONW prevalence and its epidemiological determinants in a natural population in Zhejiang province in southeastern China.

## Methods

### Study population

**Fig. 1:**
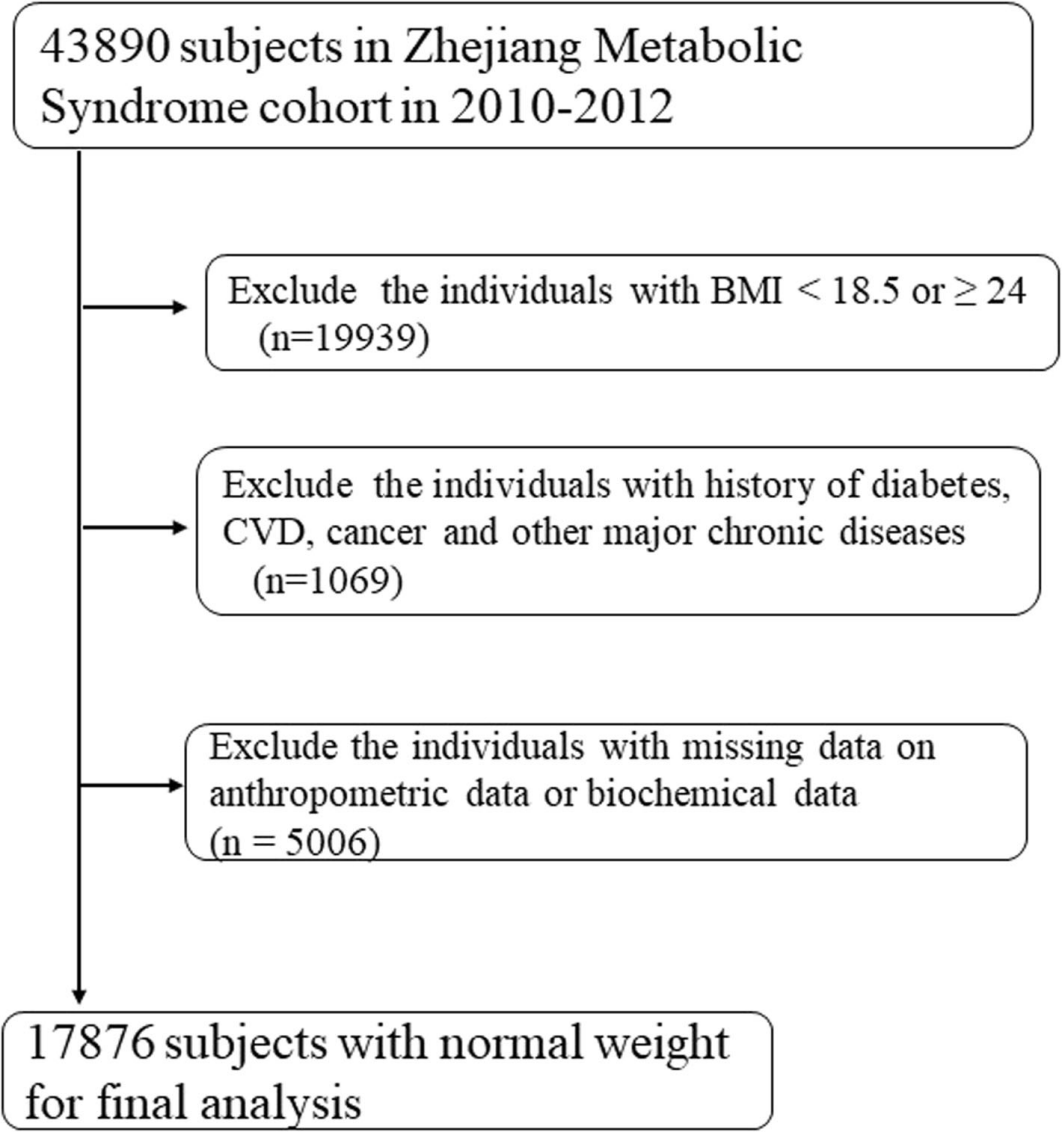
Flow chart of the study. 17,876 normal-weight individuals were recruited from 37,815 individuals in Zhejiang metabolic syndrome cohort

Individuals with normal-weight were recruited from the baseline database of the Zhejiang Metabolic Syndrome Cohort in Zhejiang province in southeastern China. The Zhejiang Metabolic Syndrome Cohort is a community-based prospective cohort study that originated from the Zhejiang Metabolic Syndrome Investigation from 2010 to 2012. The baseline investigation was a community-cluster study for the natural population in Zhejiang province. Study inclusion criteria was as follows: (1) BMI≧18.5 and <  24, (2) aged ≥20 years old, (3) Han ethnicity, and (4) lived in local community for at least two years. Individuals were excluded if they had any severe chronic diseases such as cancer, coronary heart disease, stroke, chronic cirrhosis, hyperthyroidism, or hypothyroidism. A participant recruitment flow chart is presented in Fig. 1. The study protocol was approved by the ethical review board of the Zhejiang University School of Medicine. Written informed consent was obtained from all participants.

### Anthropometric measurement

Anthropometric indexes, including weight, height, waist circumference (WC), systolic blood pressure (SBP), and diastolic blood pressure (DBP), were measured by trained investigators, following a previously described standard protocol [15]. Height and weight were measured by a scale with the participants were wearing light clothing and no shoes. WC was measured at the midpoint between the iliac crest and lowest rib. Blood pressure was measured using a mercury sphygmomanometer with the subject in a sitting position after they had rested for at least 15 min. SBP and DB*P* values were reported as the averages of three measurements with 30-s intervals.

### Biochemical determination

Whole blood and serum samples, after a 12-h overnight fast, were collected. Biochemical biomarkers, including TG, total cholesterol (TC), HDL-C and low-density lipoprotein cholesterol (LDL-C), were determined using biochemical auto-analyzers (Hitachi 7060, Tokyo, Japan). FPG was measured using a glucose oxidase method with the Beckman Glucose Analyzer (Beckman Instruments, Irvine, CA, USA).

### Epidemiological investigation

Each subject was interviewed in-person using a structured questionnaire. Information collected from participants included demographic data (e.g., date of birth, gender, educational level, and marital status), and information on smoking status (yes/no), alcohol drinking behaviors, sedentary hours, dietary behavior, and family history of diseases. Original smoking behavior in the cohort was investigated as current, former, and never. Current smoking was defined as a person smoking at least one cigarette per day for (at least) 1 year. Former smoking was defined as a person having stopped smoking for at least 1 year. In this study, former and never smokers were combined into one group. Alcohol drinking in the cohort was investigated as the frequency of drinking and was categorized into two groups ≥3 times/week and < 3 times/week. Tea drinking was classified into two groups as ≥one cup/day and < one cup/day. Menopause status was classified as a “yes” or a “no.” The time of menopause was referenced as the last natural menopause. The amount of fruit or milk intake was investigated by questionnaire-based interview and then classified into two groups, referenced as more or less, by the mean of intake amount. Food preference was investigated by questionnaire-based interview and classified as meat-based, balanced, and vegetarian, according to preference for meat and/or vegetable diets. Sedentary hours per week were calculated; participants were classified as short, moderate or long by their interquartile of sedentary hours.

### Definitions

Normal weight was defined according to criteria from the Working Group on Obesity in China (WGOC) [16] and had a body mass index (BMI) with 18.5–23.9 kg/m^2^. Metabolically abnormal traits included: (1) TG ≥1.7 mmol/L; (2) HDL-C < 1.03 mmol/L for men and < 1.29 mmol/L for women; (3) SBP ≥130 mmHg or DBP ≥85 mmHg, or using antihypertensive drug therapy; and (4) FPG ≥5.6 mmol/L or using anti-diabetic treatment. Therefore, MONW was defined as normal-weight individuals who had two or more metabolically abnormal traits. MNNW was normal-weight individuals who had one or no metabolically abnormal traits [5]. Central obesity was defined as WC > 85 cm for men and > 80 cm for women [16].

### Statistical analysis

Continuous variables with normal distribution were shown as mean and standard deviation (SD), and skewed distributions were expressed as median and interquartile range. Categorical variables were expressed as percentage (%). Student t-test was used to compare the statistical difference for continuous variables, and Chi-square tests were used for categorical variables. Multiple logistical regression models were used to calculate odds ratio (ORs) and 95% confidence intervals (CIs) after adjusting for age, gender and BMI. Metabolic status (yes/no) was the dependent variable. Age was divided into 5-year categories. BMI was analyzed according to tertiles. Waist circumference was analyzed in categories of < 70, 70–9, 80–89, and ≥ 90 cm. Additionally, Chi-square test for trend was used to analyze dose-response correlations. A two-sided *P*-value < 0.05 was considered statistically significant. All statistical analyses were performed using IBM SPSS Statistics version 22.0.

## Results

### Demographic and metabolic characteristics of the individuals

Overall, 17,876 normal-weight individuals were recruited from 37,815 individuals in the Zhejiang Metabolic Syndrome Cohort. Table 1 presents the demographic and metabolic characteristics of the individuals with MNNW and MONW. Compared to those with MNNW, the MONW population was comprised of a higher percentage of women, individuals of older age, and individuals with higher anthropometric indexes [BMI, WC, waist-hip ratio (WHR) and waist height ratio (WHtR)] and individuals with poorer metabolic traits (higher levels of blood pressure, FBG, uric acid, and dyslipidemia, and lower levels of HDL-C) and poorer liver functioning (higher levels of ALT and AST), (*P* <  0.001 for aforementioned variables).

**Table 1:** Demographic and metabolic characteristics in the individuals of metabolically normal normal-weight (MNNW), metabolically obesity normal-weight (MONW)

Variable	MNNW (*n* = 11,779)	MONW (*n* = 6097)	*P* value ^#^
Women (%)	55.4	63.6	< 0.001
Age (years)	53.9 ± 14.7	61.2 ± 11.5	< 0.001
WC (cm)	71.94 ± 15.17	76.24 ± 12.69	< 0.001
BMI	21.45 ± 1.47	22.05 ± 1.39	< 0.001
WHR	0.8 ± 0.2	0.9 ± 0.1	< 0.001
WHtR	0.45 ± 0.09	0.48 ± 0.08	< 0.001
SBP (mm·Hg)	124.1 ± 19.56	142.8 ± 19.58	< 0.001
DBP (mm·Hg)	74.8 ± 11.07	83.2 ± 11.03	< 0.001
FPG (mmol/L)	4.79 ± 0.75	5.63 ± 1.59	< 0.001
TC (mmol/L)	4.51 ± 0.94	4.81 ± 1.04	< 0.001
TG (mmol/L)	1.17 ± 0.58	2.28 ± 1.68	< 0.001
HDL-C (mmol/L)	1.57 ± 0.35	1.33 ± 0.36	< 0.001
LDL-C (mmol/L)	2.43 ± 0.73	2.54 ± 0.77	< 0.001
ALT (U/L)	18 (14–23)	19 (15–26)	< 0.001
AST (U/L)	23 (19–28)	24 (21–30)	< 0.001

1. Normal-weight was defined as 18.5 ≤ BMI <  24 kg/m2. Metabolic normality was defined as the individuals with ≤1 of metabolic abnormality in blood pressure, FPG, TG and HDL-C

2. Data are presented as mean ± standard deviation except sex with percentage

3. *Abbreviation*: *MNNW* Metabolically normal normal-weight, *MONW* Metabolically obesity normal-weight, *WC* Waist circumference, *BMI* Body mass index, *SBP* Systolic blood pressure, *DBP* Diastolic blood pressure, *FPG* Fasting plasma glucose, *TC* Total cholesterol, *TG* Triglycerides, *HDL-C* High density lipoprotein cholesterol

4. # Comparisons between MNNW and MONW

### Prevalence distributions of MONW by gender and age

**Fig. 2:**
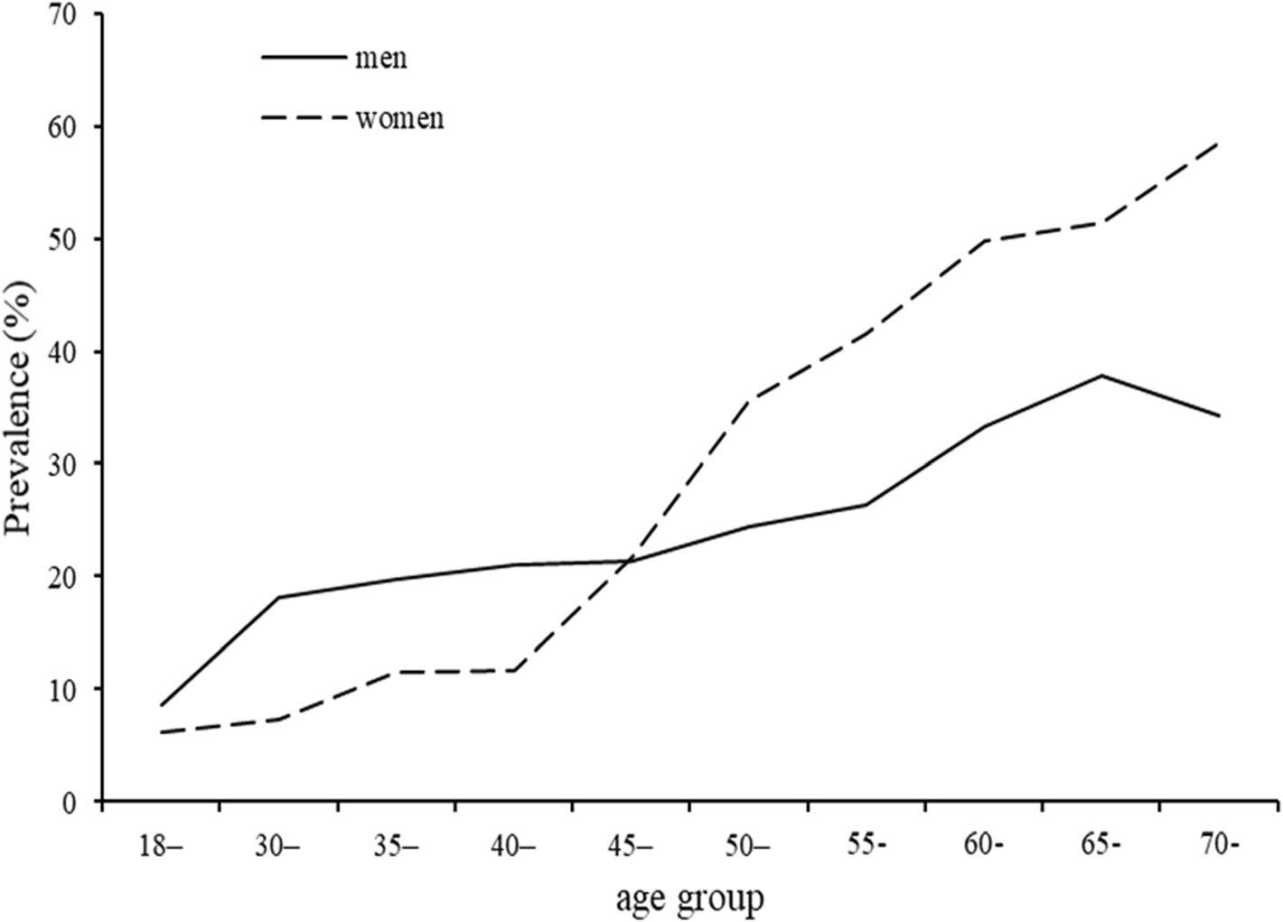
the prevalence distribution of MONW by men and women. The prevalence of MONW positively correlated with age. Different distributions of MONW were found between males and females depending on age group. For the individuals aged < 45 years old, man had significantly higher prevalence of MONW than women, while for the individuals aged ≥50 years, men had significantly lower prevalence than women

Among 17,876 normal-weight individuals, 6097 were deemed metabolic obese, and the MONW prevalence rate was 34.1% in normal-weight individuals and 16.1% for the general population. The overall MONW prevalence in men was significantly lower than the prevalence in women (29.7% vs. 37.3%, *P* <  0.001), with an overall men/women ratio of 0.8. However, the different distribution of MONW prevalence between men and women varied by age. For instance, among individuals aged < 45 years old, men had a significantly higher prevalence than women (men/women ratio > 1, and all the *P* values < 0.01), except for the group aged 18–29 years old (*P* = 0.167). No statistically significant differences were found for the 45–49-year-old age group (*P* = 0.886). Among individuals aged ≥50 years old, men had a significantly lower prevalence than women (men/women ratio < 1, and all the *P* values < 0.001) (Table 2 and Fig. 2).

**Table 2:** Prevalence of MONW of the individuals stratified by age and gender

	All individuals (*n* = 17,876)	males (*n* = 7475)	females (*n* = 10,401)	*P* value *	Ratio of prevalence
Overall	6097 (34.1)	2220 (29.7)	3877 (37.3)	< 0.001	0.80
Age group					
20-	66 (7.0)	29 (8.6)	37 (6.2)	0.167	1.39
30-	64 (11.2)	37 (18.1)	27 (7.3)	< 0.001	2.48
35-	120 (15.2)	66 (20.4)	54 (11.6)	0.001	1.76
40-	163 (16.0)	91 (21.6)	72 (12.1)	< 0.001	1.78
45-	302 (22.0)	106 (21.8)	196 (22.1)	0.886	0.99
50-	776 (33.4)	138 (25.0)	638 (36.0)	< 0.001	0.69
55-	1136 (37.4)	258 (27.0)	878 (42.2)	< 0.001	0.64
60-	1333 (42.1)	555 (33.9)	778 (51.0)	< 0.001	0.66
65-	838 (45.4)	395 (38.6)	443 (53.7)	< 0.001	0.72
70-	1299 (46.3)	545 (35.6)	754 (59.0)	< 0.001	0.60
*P*_trend_	< 0.001	< 0.001	< 0.001		

Data are presented as the number and percentage (%) of MONW

* Prevalence comparison of MONW between males and females

The MONW prevalence of was positively correlated with age for both men and women (*P*_trend_ = 0.001). Among normal-weight individuals aged ≧60 years, 54.4% of women and 35.7% of men with normal-weight were MONW (Table 2).

### The risk factors associated with MONW in normal-weight individuals

Using a multiple logistic regression model and adjusting for confounding effects by age and gender, we examined the associations between epidemiological factors and metabolic status in normal-weight individuals. To examine whether these associations were independent of BMI, we also adjusted for BMI besides age and gender. As shown in Table 3, BMI was positively associated with MONW risk, even among individuals with a normal BMI. Compared to individuals with the lowest BMI tertile of 18.5–20.8 kg/m^2^, individuals with a BMI tertile of 20.9–22.4 kg/m^2^ had a higher MONW risk (OR = 1.63, 95% CI: 1.45–1.83) as did individuals with a BMI tertile of 22.5–24.0 kg/m^2^ (OR = 2.68, 95% CI: 2.40–3.00). A positive relationship was found between BMI levels and MONW risk (*P*_trend_ <  0.001). Higher WC levels were also associated with increased MONW risk. Compared to individuals with WC < 70 cm, individuals with WC > 70 cm had a significantly more elevated MONW risk, with ORs of 1.60 (95% CI: 1.40–1.82) for WC 70–79 cm, 3.06 (95% CI: 2.64–3.54) for WC 80–89 cm, and 4.27 (95% CI: 2.95–6.19) for WC > 90 cm. WC was also positively correlated with MONW risk (*P*_trend_ <  0.001). Importantly, these associations were still statistically significant after additional adjustment for BMI. Further, central obesity was significantly associated with increased MONW risk with the ORs reaching 2.19 (95% CI: 1.92–2.50) and 1.58 (95% CI: 1.38–1.82) with and without the additional adjustment for BMI besides sex and age, respectively. Individuals with a family history of diseases, such as diabetes, cardiovascular diseases had an increased MONW risk, with an OR of 1.52 (95%CI: 1.32–1.75). Furthermore, women with menopause, compared to those without menopause, had an increased MONW risk, with an OR of 2.06 (95% CI: 1.66–2.54) after adjusting for age and BMI.

**Table 3:** The risk factors associated with metabolic abnormality in the normal-weight individuals

Variables	MNNW (11779)	MONW (6097)	OR^a^	OR^b^
BMI				
18.5-	4437 (37.7)	1350 (22.1)	Reference	
20.9-	3979 (33.8)	1997 (32.8)	1.69 (1. 55–1.84)	
22.5–24.0	3363 (28.6)	2750 (45.1)	2.79 (2.57–3.03)	
			< 0.001	
Waist circumference (cm)				
< 70	2576 (24.6)	586 (11.3)	Reference	Reference
70-	5636 (53.8)	2586 (49.7)	1.72 (1.55–1.92)	1.33 (1.19–1.48)
80-	2136 (20.4)	1852 (35.6)	3.35 (2.98–3.77)	2.05 (1.79–2.34)
≥ 90	120 (1.1)	182 (3.5)	5.62 (4.33–7.28)	3.29 (2.51–4.32)
P trend			< 0.001	< 0.001
Central obesity^c^				
No	10,739 (91.6)	4957 (81.3)	Reference	Reference
Yes	986 (8.4)	1140 (18.7)	2.03 (1.84–2.23)	1.44 (1.31–1.60)
Education level				
Primary school or below	1262 (39.4)	886 (60.4)	Reference	Reference
Secondary school	1558 (48.7)	541 (36.9)	0.73 (0.63–0.84)	0.71 (0.62–0.83)
College or upper	382 (11.9)	41 (2.8)	0.30 (0.21–0.43)	0.30 (0.21–0.42)
Smoking status				
(former and never)	5449 (74.2)	2711 (81.3)	Reference	Reference
current	1893 (25.8)	623 (18.7)	0.90 (0.79–1.03)	0.92 (0.80–1.05)
Regular alcohol drinking				
No < 3 times/week	4353 (62.3)	2295 (69.7)	Reference	Reference
≥ 3 times/week	2631 (37.7)	999 (30.3)	0.85 (0.77–0.94)	0.83 (0.74–0.91)
Regular tea drinking				
< one cup/day	5633 (68.3)	2762 (74.0)	Reference	Reference
≥ one cup/day	2614 (31.7)	969 (26.0)	1.03 (0.93–1.14)	1.00 (0.90–1.11)
Sedentary time^d^				
Short (< 2 h)	746 (13.6)	331 (13.6)	Reference	Reference
Moderate or longer (≥ 2 h)	4742 (86.4)	2101 (86.4)	1.00 (0.87–1.16)	1.03 (0.89–1.19)
food preferences				
Meat-based	253 (9.9)	164 (14.2)	Reference	Reference
balanced	1391 (54.5)	503 (43.6)	0.57 (0.45–0.72)	0.59 (0.46–0.75)
vegetarian	907 (35.6)	487 (42.2)	0.62 (0.49–0.80)	0.65 (0.51–0.83)
Fruit intake ^e^				
Less	616 (67.3)	466 (72.0)	Reference	Reference
More	299 (32.7)	181 (28.0)	0.95 (0.75–1.20)	0.95 (0.75–1.21)
Milk intake ^e^				
Less	262 (75.1)	152 (74.1)	Reference	Reference
More	87 (24.9)	53 (25.9)	1.12 (0.74–1.70)	1.11 (0.72–1.70)
Family history of diseases ^f^				
No	1191 (46.0)	552 (40.4)	Reference	Reference
Yes	1399 (54.0)	816 (59.6)	1.52 (1.32–1.75)	1.45 (1.26–1.67)
Menopause				
No	856 (45.4)	199 (16.8)	Reference	Reference
Yes	1029 (51.1)	985 (48.9)	2.10 (1.67–2.65)	2.04 (1.62–2.58)

OR^a^, odds ratio after adjusting for age and gender. OR^b^, odds ratio after adjusting for age, gender, BMI, and WC

^c^, evaluated based on waist circumference > 85 cm for males and 80 cm for females

^d^Sedentary time were classified as short, moderate and long by the interquartile of sedentary hours

^e^ More and less of the dietary intakes are defined by the mean cut-points of the amount of the dietary intakes per week

^f^ Family history of diseases includes diabetes, cardiovascular diseases

Higher education level, regular alcohol drinking, and balanced or vegetarian food preferences were significantly associated with better metabolic profiles in normal-weight individuals. Compared with individuals with only a primary school education, individuals with a college or higher education had a reduced MONW risk, with ORs of 0.30 (95% CI: 0.21–0.42). Regular alcohol drinking was associated a decreased MONW risk (OR = 0.79, 95% CI: 0.68–0.92). Individuals with balanced and vegetarian food preferences had a reduced MONW risk, with ORs of 0.59 (95% CI: 0.46–0.75) and 0.65 (95% CI: 0.51–0.83), respectively. No statistically significant associations were found in daily intakes of vegetables, fruit, or milk, tea drinking, or sedentary time (all the *P* values > 0.05).

## Discussion

In this study, we found that the overall MONW prevalence was 34.1% in normal-weight individuals, and was 16.1% in the general population. The MONW distribution between men and women varied by age. Education level, alcohol drinking behavior, balanced or vegetarian food preferences, menopause status, and family history of diseases, were all associated with metabolic status in the normal-weight population.

The MONW prevalence around the world varies greatly [4, 6, 13, 14, 17]. Previous studies have indicated that the global MONW prevalence ranges from as low as 5% to as high as 45%. A meta-analysis by Wang et al. [17] estimated that the overall global MONW prevalence in normal-weight individuals is 30% (95% CI: 26–36%); however, the authors found high heterogeneity in prevalence among the analyzed studies. This prevalence variation may be due to individuals’ age, gender, ethnicity, geographic location, and MONW definitions (i.e., criteria for obesity and metabolically abnormal, respectively). Metabolic parameters such as metabolic traits, insulin resistance, and subclinical inflammation have been widely used to define metabolic abnormality [18]. However, in most circumstances, insulin resistance and subclinical inflammation are not determined, therefore, an individual may be practically considered metabolically healthy when fewer than two parameters of metabolic syndrome are abnormal [8, 19, 20]. In this study, with this common criterion for metabolic health employed, the MONW prevalence was 34.1% in normal-weight individuals. Obesity was defined as BMI 18.5–23.9 kg/m^2^ and metabolic abnormality referenced at least two abnormal traits among the factors of TG, HDL-C, BP and FPG. Using the same definition of metabolic risk, the Multi-Ethnic Study of Atherosclerosis showed high variability in the prevalence of metabolically unhealthy with normal-weight individuals based on ethnicity: The prevalence was 21.0% in Whites, 32.2% in Chinese Americans, 31.1% in African Americans, 38.5% in Hispanics, and 43.6% in South Asians [21]. The prevalence among Chinese was highly consistent between our findings in mainland China and the findings in America. Although there are many criteria to evaluate MONW, currently, no consensus has been reached to define the MONW syndrome, thus comparisons of prevalence should be cautious in considering results from different studies.

Age and gender were associated with MONW prevalence, with age shown to be consistently positively correlated with prevalence; individuals with MONW were generally older (aged > 50 years). Therefore, even in the normal-weight individuals, metabolic abnormality may be exacerbated with increasing age. However, we found different distributions between men and women. In one study, male gender was found to be a MONW risk factor [22], while females ha ad higher MONW prevalence in another study [14]. This inconsistency could be well explained by our findings. We found that the gender-specific prevalence was associated with age. The age range of 45–49 years old was a crossover point between men and women for MONW prevalence changing with age (Table 2). This distribution was similar with metabolically normal obesity in our previous study [15]. We also found that menopause increased MONW risk (having adjusted for the confounding effect by age). Therefore, combining these results, it appears that estrogen may be a protective factor for maintaining metabolic health in normal-weight females [23, 24].

Previous studies have reported that low physical activity levels and high sedentary behavior levels are associated with increased risks of metabolic abnormality and cardio-metabolic diseases [25, 26]. However, in the present study, no statistically significant association was found between sedentary time and metabolic status in normal-weight individuals. Additionally, Dunstan et al. found a deleterious effect of TV watching time on abnormal glucose metabolism risk [27]. In China, TV watching time is a major component in sedentary time (besides sleeping time). This association should be further researched in future scholarship. It has been shown that physical activity promotes metabolic health and reduces the risk of diabetes and cardiovascular diseases even among normal-weight individuals.

Daily diet was associated with metabolic status. A healthy diet helps maintain metabolic normality in both obese [15, 28] and normal-weight persons. This study also found that regular alcohol drinking, and balanced or vegetarian food preferences might reduce MONW risk. Previous studies have found that normal-weight individuals who drank alcohol had a slightly higher metabolic abnormality prevalence, which was not consistent with our findings [17]. The inconsistent results may be attributable to the difference in alcohol intake between Chinese and Western populations. Chinese have a relatively lower amount of alcohol intake compared to the Western population [29]. It has been reported that the dose of alcohol is associated with the biological effect on diabetes and cardiovascular diseases [30]. Thus, it is believed that lower alcohol intake may be beneficial to maintaining metabolic health and reducing cardiovascular disease risk [31]. No statistically significant association was found between cigarette smoking and metabolic status in normal weight individuals. This result was consistent with our previous study examining metabolic status in obesity [15]. However, inconsistent findings were found in previous studies [32].

Family history is also a risk factor for metabolic status. A family history of hypertension was significantly more common in MONW individuals than in MNNW individuals [22]. Though there was no statistically significant difference found in individuals with a family history of obesity, dyslipidemia, type 2 diabetes, or coronary heart disease, the percentage of family histories tended to be higher in MONW individuals than in MNNW individuals in previous studies [33, 34]. The above results may be due to the small sample size and low statistical power. In our study, using relatively large samples, we found that family histories of diabetes, and cardiovascular diseases increased MONW risk. The individuals with the same family history may have common genetic factors and/or common environmental factors such as dietary or physical activity behavior. Previous studies have revealed that 10 variants (in or near *IRS1*, *GRB14*, *ARL15*, *PPARG*, *PEPD*, *ANKRD55*/*MAP 3 K1*, *PDGFC*, *LYPLAL1*, *RSPO3*, and *FAM13A1*) are associated with metabolic status [35, 36]. These findings indicate that genetic susceptibility might play an important role in health abnormality in normal-weight individuals.

Body mass is regarded as an important determinant of obesity-related diseases. A BMI of 18.5–24.0 kg/m^2^ is associated with the lowest all-cause mortality [37]. Maintaining a BMI in this range may effectively reduce the risk of diabetes, cardiovascular diseases and premature death [38]. We found that BMI was also positively correlated with metabolic abnormality risk, even in the normal BMI range. Under the same BMI levels, compared with Americans, Chinese have more metabolic abnormality in BP, TG, and HDL-c, etc., and may have a higher body fat percentage among normal-weight individuals [39]. Therefore, a lower BMI threshold is suggested in weight evaluation, especially for older persons [7, 40].

WC significantly correlated with metabolic abnormality risk even after adjustment for BMI. Central obesity in normal-weight individuals was also found to increase metabolic abnormality risk. These findings indicate that central obesity is an independent risk factor for metabolic status. A total of 27.0% of individuals with normal weight had central obesity [41]. Although BMI remains a useful index to assess overweight and obesity, it has limitations in differentiating body fat from lean mass, and central fat from peripheral fat [6]. Athletes, who have high muscle mass and low body fat, may be misclassified using BMI [42]. Asians with small builds have relatively low fat-free mass and high body fat [7, 43]. Therefore, central obesity-associated indices such as WC, waist-hip ratio, and waist height ratio may be better markers for obesity evaluation.

This study has some strengths. The epidemiological data were collected by trained health professionals, and the biochemical measurements were followed by standard protocol. To increase the comparability of the results, we used the most common definitions of normal weight and metabolic abnormality. However, there were several limitations to this study. Participants were recruited from the Zhejiang Metabolic Syndrome Cohort, and some individuals were excluded because of missing data on BMI and metabolic parameters. These excluded individuals could have induced selection bias and thus increase the difficulty in extrapolating our findings. Further, the study was cross-sectional. Associations from this research should be further examined by future prospective study.

## Conclusions

This study found that there is metabolic heterogeneity in normal-weight individuals in China. The prevalence of metabolic abnormality is 34.1% in normal-weight individuals and 16.1% in the general population. MONW distribution between men and women varies by age. BMI, WC, education level, alcohol drinking, balanced or vegetarian food preferences, and menopause status, and family histories of diabetes, cardiovascular diseases, are associated with the metabolic status in normal-weight individuals. These findings provide new evidence for management of normal-weight individuals and have important public health implications.

## Data Availability

This is an ongoing project. The datasets used and/or analysed during the current study are available from the corresponding author and investigating coordinators on reasonable request.

## Abbreviations

MONW: Metabolically obesity normal-weight
BMI: Body mass index
WC: Waist circumference
CHD: Coronary heart disease
FPG: Fasting plasma glucose
HDL-C: High-density lipoprotein cholesterol
SBP: Systolic blood pressure
DBP: Diastolic blood pressure
LDL-C: Low-density lipoprotein cholesterol
WHR: Waist-hip ratio
WHtR: Waist height ratio
